# Rational Mutagenesis in the Lid Domain of Ribokinase from *E. coli* Results in an Order of Magnitude Increase in Activity towards D-arabinose

**DOI:** 10.3390/ijms232012540

**Published:** 2022-10-19

**Authors:** Evgeniy A. Zayats, Ilya V. Fateev, Maria A. Kostromina, Yulia A. Abramchik, Dmitry D. Lykoshin, Daria O. Yurovskaya, Vladimir I. Timofeev, Maria Ya. Berzina, Barbara Z. Eletskaya, Irina D. Konstantinova, Roman S. Esipov

**Affiliations:** Shemyakin and Ovchinnikov Institute of Bioorganic Chemistry, Russian Academy of Sciences, Miklukho-Maklaya St. 16/10, 117997 Moscow, Russia

**Keywords:** enzyme genetic improvement, rational design, active site modification, site-directed mutagenesis, ribokinase, cascade synthesis, modified nucleoside, arabinoside, arabinose

## Abstract

Development of efficient approaches for the production of medically important nucleosides is a highly relevant challenge for biotechnology. In particular, cascade synthesis of arabinosides would allow relatively easy production of various cytostatic and antiviral drugs. However, the biocatalyst necessary for this approach, ribokinase from *Escherichia coli* (*Eco*RK), has a very low activity towards D-arabinose, making the synthesis using the state-of-art native enzyme technologically unfeasible. Here, we report the results of our enzyme design project, dedicated to engineering a mutant form of *Eco*RK with elevated activity towards arabinose. Analysis of the active site structure has allowed us to hypothesize the reasons behind the low *Eco*RK activity towards arabinose and select feasible mutations. Enzyme assay and kinetic studies have shown that the A98G mutation has caused a large 15-fold increase in k_cat_ and 1.5-fold decrease in K_M_ for arabinose phosphorylation. As a proof of concept, we have performed the cascade synthesis of 2-chloroadenine arabinoside utilizing the A98G mutant with 10-fold lower amount of enzyme compared to the wild type without any loss of synthesis efficiency. Our results are valuable both for the development of new technologies of synthesis of modified nucleosides and providing insight into the structural reasons behind *Eco*RK substrate specificity.

## 1. Introduction

One of the key challenges of modern biotechnology is the development of efficient approaches for the synthesis of medically important drugs. A noteworthy class of compounds that are broadly applied for treatment of a large variety of diseases are modified nucleosides [1]. These compounds act as mimics of their natural nucleoside and nucleotide counterparts, therefore interfering in the biological processes that are dependent on DNA and RNA metabolism [2]. Such biological activity allows these compounds to suppress the proliferation of tumor cells, bacteria and viruses, therefore resulting in broad-spectrum activity against cancer and infectious diseases. Therefore, the development of new and efficient technologies for the production of modified nucleosides is an important scientific task.

Conventional approaches involving chemical synthesis remain a laborious multi-stage process requiring the introduction and removal of protective groups, in some cases resulting in poor product yield and limited process efficiency [1,3]. As an alternative, cascades of enzymes involved in nucleic acid metabolism offer single-step one-pot synthesis of nucleosides and nucleotides with high stereo- and regioselectivity [1,3,4,5,6]. Nevertheless, one of the key factors limiting the efficiency of the cascade synthesis of modified nucleosides is the low activity of the wild type enzymes towards the necessary unnatural substrates.

**Figure 1:**
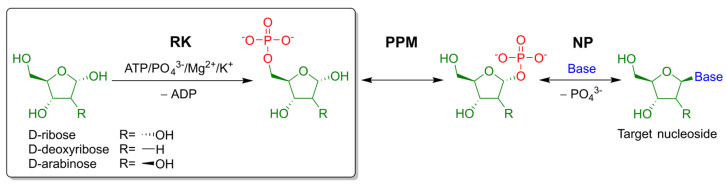
A three-enzyme cascade for the synthesis of modified nucleosides. RK—ribokinase (ec:2.7.1.15), PPM—phosphopentomutase (ec: 5.4.2.7), NP—nucleoside phosphorylase, usually purine nucleoside phosphorylase (ec:2.4.2.1). Base—nucleobase substrate/nucleoside moiety.

On one hand, enzymes that carry out the glycosylation reaction in the cascade synthesis of nucleosides tend to have broad substrate specificity towards the nucleobase substrates [7,8,9]. On the other hand, enzymatic synthesis of nucleosides with a modified sugar moiety turns out to be more challenging. Sugar-modified nucleosides availability is strictly limited. Therefore, these compounds cannot be obtained from commodity reagents through nucleoside phosphorylase mediated transglycosilation. To overcome this challenge, a three-enzyme (ribokinase, phosphopentomutase and nucleoside phosphorylase) cascade was developed, offering a synthesis starting from easily accessible pentoses (Figure 1) [4,10].

**Figure 2:**
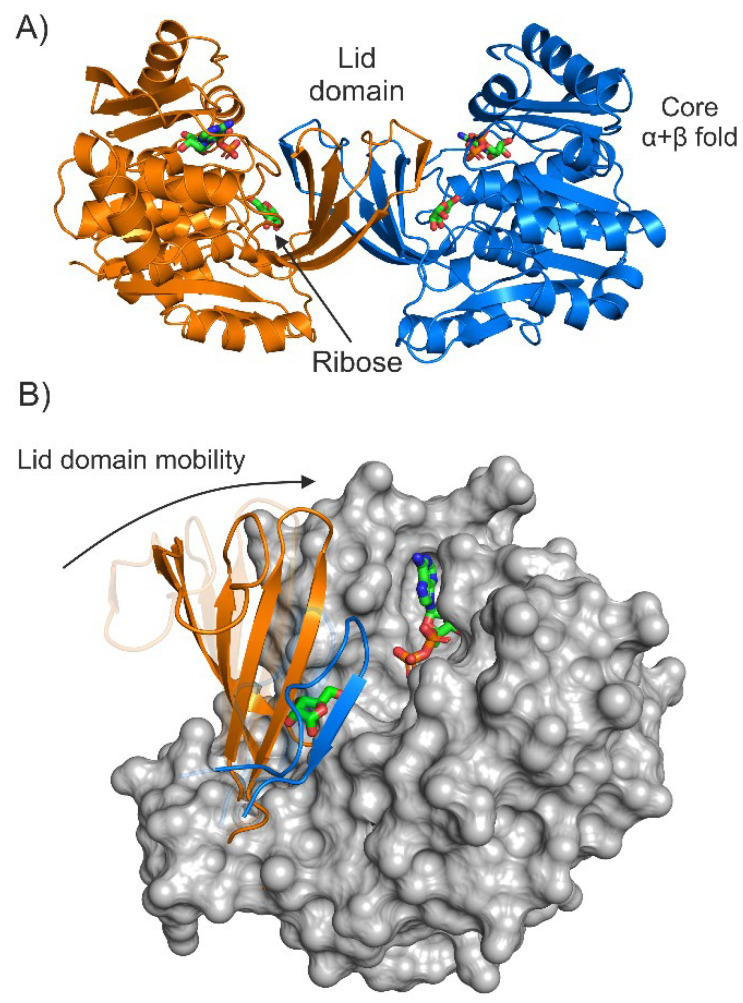
(**A**)—The crystal structure of the *Eco*RK dimer (PDB 1RKD). (**B**)—The mobility of the lid domain during the transition from APO form (semi-transparent, spatial alignment with crystal structure of APO *Eco*RK, PDB 1RKA) to substrate-bound form (PDB 1RKD). The lid domain is represented as cartoon, all other residues—as a surface.

The first enzyme of this cascade is ribokinase (ec:2.7.1.15). This enzyme catalyzes the phosphorylation of D-ribose through the 5′-hydroxyl group, using ATP as the phosphate donor (Figure 1, first reaction). Ribokinase from both *E. col*i and other sources is a homodimer with two symmetrical active sites [11,12,13,14]. Each subunit contains two functional domains: the core α + β fold and the so-called lid domain (Figure 2A) [11]. The lid domain is a five-stranded β-sheet, formed through cooperation between enzyme subunits: four strands come from one subunit and one from another.

The transition of *Eco*RK from APO to a substrate-bound form is accompanied by a significant conformational change due to the lid-domain mobility. The mentioned 5-stranded β-sheet acts as a lid (hence the name) that is open in APO form and closed upon ribose binding (Figure 2B) [11,12]. Ribose binding occurs through a network of hydrogen bonds between the hydroxyl carbohydrate groups and the active site residues: Asn14, Asp16, Gly42, Lys43, Asn46, Glu143 and Asp255. A residue interacting with the 5′-hydroxyl group of ribose, Asp255 is believed to be catalytic and responsible for the deprotonation of this group, preceding the formation of an ester with a phosphate [11].

The ability of ribokinase to phosphorylate pentoses allows the introduction of carbohydrates (including those that are not present as moieties of natural nucleosides) into enzymatic cascade-reaction pathways. Therefore, utilization of this enzyme is a valuable alternative to transglycosylation for the synthesis of nucleosides with unnatural pentose moieties. Unfortunately, the substrate specificity of ribokinase is relatively narrow. We have previously shown that for both ribokinase from *Escherichia coli* (*Eco*RK) and *Thermus sp. 2.9* (*Tsp*RK), only ribose and deoxyribose are good substrates [15,16].

One of the most medically important classes of sugar-modified nucleoside analogues are the derivatives of D-arabinose. Noteworthy broadly applied arabinoside drugs include compounds with both cancer suppressing (fludarabine, nelarabine, cytarabine) and antiviral (vidarabine) activity [17,18,19,20]. Our previous studies show that arabinose is a substrate for both *Eco*RK and *Tsp*RK, although the enzymatic activity towards this pentose is very low [4,15,16]. According to Park et al., the human ribokinase has a very weak activity towards arabinose as well [21]. We have previously shown that the wild type ribokinases from both *E. coli* and *T. sp.* can be used for cascade synthesis of arabinonucleosides [4,22]. However, even at a very high excess of arabinose, a large amount of the recombinant enzyme was still necessary for efficient synthesis. Therefore, the feasibility of industrial cascade synthesis of arabinonucleoside drugs using the state-of-art wild type enzymes remains highly questionable.

To summarize, the narrow carbohydrate substrate specificity of ribokinase limits its applicability for the synthesis of a variety of medically important arabinonucleoside drugs. A rapidly developing approach, offering an opportunity to overcome this challenge is enzyme design. An absent but desired property of a biocatalyst (in our case, substrate promiscuity) can be engineered through the study of the enzyme active site and subsequent rational modification via site-directed mutagenesis. In our research piece, we report the successful rational design of a mutant form of ribokinase from *Escherichia coli* with a significant increase in turnover rate (k_cat_) and decrease in K_M_. We have chosen this enzyme instead of *Tsp*RK since *Eco*RK has a broad temperature range of activity, allowing to design cascades with enzymes from both mesophilic and thermophilic sources [4]. Our research is significant both for developing new technologies for the cascade synthesis of medically important compounds and for the study of the relationship between ribokinase active site structure and function.

## 2. Results

### 2.1. Selection of Design Hot Spots and Feasible Mutations

The first stage of our enzyme design project was to study the structure of the active site of ribokinase from *Escherichia coli* in order to hypothesize the factors determining its substrate specificity, select hotspots for enzyme design and choose mutations beneficial for arabinose binding.

**Figure 3:**
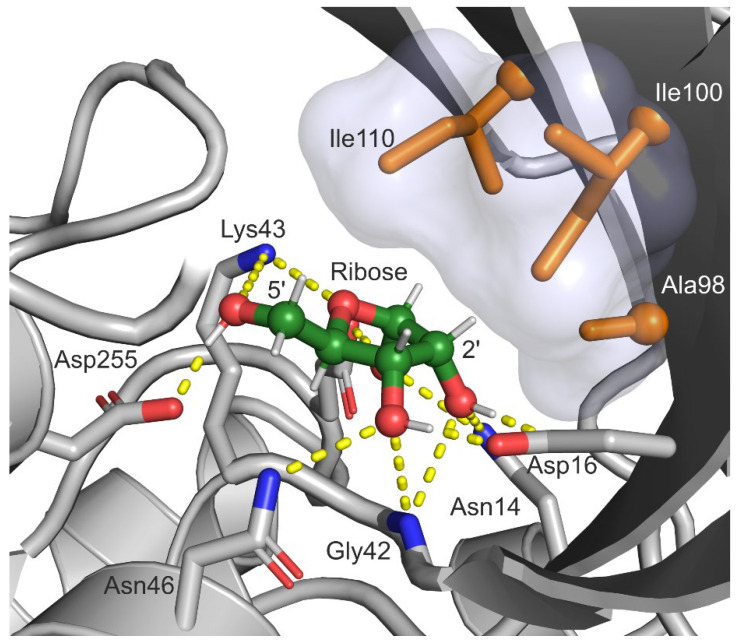
The position of ribose in the active site of *Eco*RK (PDB 1RKD). The semi-transparent surface represents the hydrophobic interface in the lid domain, formed by Ala98, Ile100 and Ile110. Residues 248–251 and 285–295 are hidden. Carbon atoms of ribose are colored green, carbon atoms of residues Ala98, Ile100 and Ile110—orange, oxygen atoms—red, nitrogen atoms—blue. Yellow dashed lines represent polar contacts Visualization was performed in PyMOL 2.0 (Schrödinger, LLC).

Our compound of interest, arabinose, is a stereoisomer of the natural substrate ribose. The only difference between these molecules is the chirality of the 2′ carbon (C2′) atom. Therefore, if we inverse the chirality of this atom of ribose in the crystal structure of *Eco*RK, we can make an initial estimation of the position of arabinose in the active site. On Figure 3, we can observe that ribose assumes the O4’-endo conformation in the active site of *Eco*RK (PDB 1RKD). We could hypothesize that arabinose binds to *Eco*RK in a way that is similar to the natural substate. In order to verify this assumption, we have studied the binding of ribose and arabinose by *Eco*RK in silico.

To perform a comparative study of ribose and arabinose binding in the active site of *Eco*RK, we have carried out molecular dynamics (MD) simulations in Gromacs 2021 using the Amber AMBER99SB-ILDN forcefield. The structure of *Eco*RK in complex with the natural substrate was fetched from Protein Data Bank (PDB 1RKD). Complex with arabinose (initial approximation) was obtained by reversing the chirality of the C2′ ribose atom through molecular editing. Rhombic dodecahedral periodic cells with protein-ligand complexes were filled with tip3p water-molecule models, neutralized with 150 mM KCl ions and subjected to energy minimization, followed by NVT and NPT equilibration. The final step was productive MD, which resulted in two 400-ns long MD simulation trajectories.

**Figure 4:**
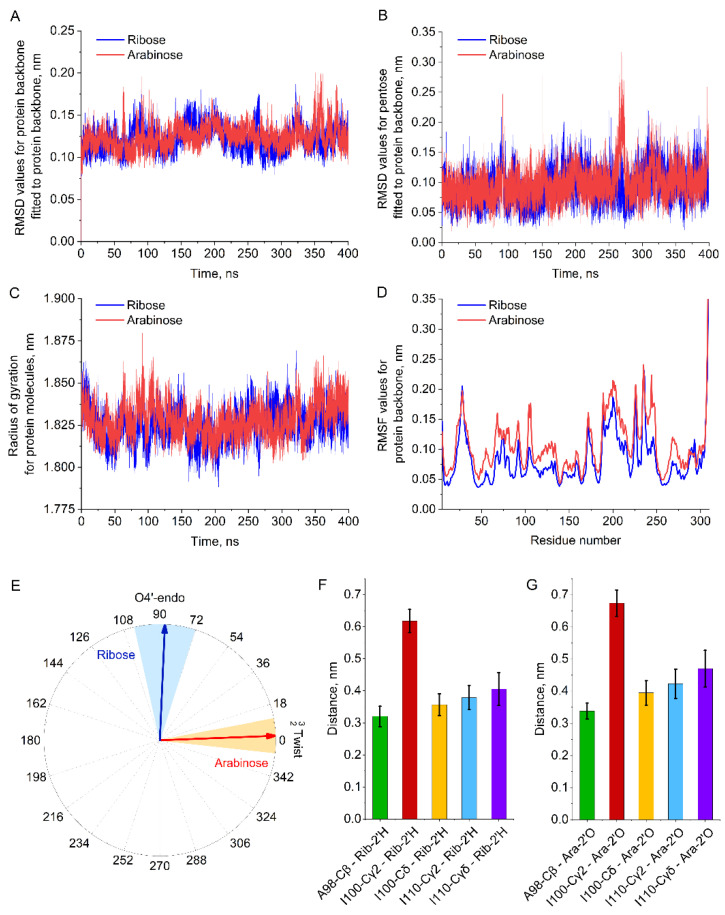
(**A**)—RMSD values for protein backbone fitted to protein backbone (**B**)—RMSD values for pentose molecule fitted to protein backbone (**C**)—radius of gyration for the protein molecule (**D**)—RMSF profile for the protein backbone (**E**)—pseudorotation coordinates for the furanose ring of ribose/arabinose (mean value and standard deviation) (**F**)—Distances between 2′-hydrogen atom of ribose and various atoms of *Eco*RK active site (mean values and standard deviation) (**G**)—Distances between 2′-oxygen atom of arabinose and various atoms of *Eco*RK active site (mean values and standard deviation).

An analysis of the obtained trajectories (RMSD, RMSF and radius of gyration) verified the stability of the complex between *Eco*RK and both the natural and unnatural substrate throughout the MD simulations (Figure 4A–D). Therefore, the mentioned MD simulation trajectories are appropriate for further analysis.

**Figure 5:**
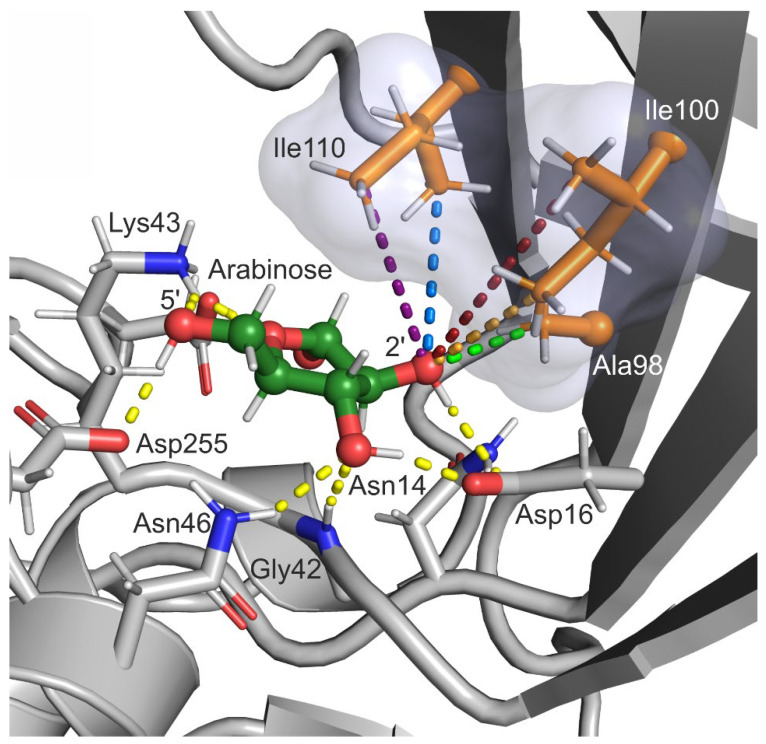
The position of arabinose in the active site of *Eco*RK (structure obtained through cluster analysis of 400-ns long MD trajectory). The semi-transparent surface represents the hydrophobic interface in the lid domain, formed by Ala98, Ile100 and Ile110.Residues 248–251 and 285–295 are hidden. Carbon atoms of arabinose are colored green, carbon atoms of residues Ala98, Ile100 and Ile110—orange, oxygen atoms—red, nitrogen atoms—blue. Yellow dashed lines represent polar contacts; other colors distances measured throughout the MD simulation and represented in Figure 4F,G. Visualization was performed in PyMOL 2.0 (Schrödinger, LLC).

The conformation of the active site of *Eco*RK in complex with arabinose (obtained from cluster analysis) is illustrated in Figure 5. We can observe that most of the interactions between arabinose and the active site are similar to those of the natural substrate (Figure 3). However, calculation of furanose ring pseudorotation coordinates via PLUMED has revealed that arabinose assumes the  Twist23 puckering conformation, unlike the O4′-endo envelope of ribose (Figure 4E). Therefore, the experiment in silico has shown that while the interactions of ribose and arabinose with active site residues are similar, the conformation of the ligand itself is different.

One of the main differences between ribose and arabinose interactions with the active site residues is that the Asn14 residue does not form a hydrogen bond with arabinose, which may be a factor hindering *Eco*RK activity towards the unnatural substrate. Nevertheless, according to Kang et al., mutation of a homologous Asn78 residue to alanine in ribokinase from Arabidopsis thaliana did not result in a significant decrease in activity towards ribose [23]. This fact suggests that the loss of aforementioned hydrogen bond may not be critical for pentose phosphorylation. Therefore, investigation of other factors is a more feasible research direction.

Further comparative analysis of the positions of arabinose and ribose in the active site of *Eco*RK allows us to determine another factor that may play a role in the enzyme’s substrate specificity. In Figure 3 and Figure 5, we can observe that the residues Ala98, Ile100 and Ile110 form a hydrophobic interface in the lid domain. In the case of the natural substrate, this hydrophobic interface is exposed to the non-polar 2′ and 3′ hydrogen atoms of ribose (Figure 3). However, in the case of our compound of interest, the same interface is exposed to the polar 2′-hydroxyl group of arabinose (Ara-2′OH), potentially resulting in a unfavorable interaction. Therefore, we can hypothesize that one of the reasons behind the low affinity of *Eco*RK towards arabinose is a steric conflict between Ara-2′OH and the hydrophobic interface of the lid domain formed by Ala98, Ile100 and Ile110. We have decided to focus our enzyme design project on the solution of this possible issue, aiming both to test our hypothesis experimentally and, hopefully, obtain the desired mutant *Eco*RK with elevated affinity towards arabinose.

Throughout the MD simulations, the atoms of the three mentioned lid-domain residues remained at the same distance from both 2′-oxygen atom of arabinose and 2′-hydrogen atom of ribose (Figure 4F,G). The closest atom to the 2′-oxygen atom of arabinose was the β-atom of Ala98, followed by the δ-atom of Ile100. Therefore, the two most promising mutations are A98G and I100V. There might be a possibility that these substitutions may act in a synergetic manner, while having no effect individually, making the double A98G/I100V mutant also potentially feasible. In order to study the role of the third mentioned residue, the I110V is also worthy of experimental investigation. Further reduction in the Ile sidechain (mutations to Ala or Gly) may not be feasible before we test the less disruptive substitutions.

To conclude, investigation of the *Eco*RK active site in silico has allowed us to hypothesize the reasons behind low affinity of this enzyme towards arabinose and select three hot spots for enzyme design with four mutations worthy of experimental study: A98G, I100V, I110V and A98G/I100V.

### 2.2. Enzyme Assay, Kinetic Studies and Cascade Synthesis

**Figure 6:**
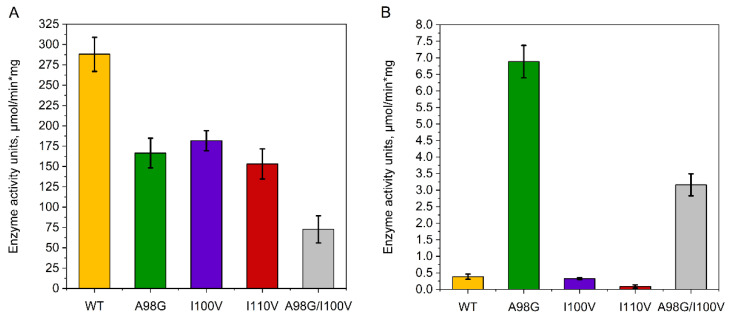
Enzyme assay of wild type *Eco*RK and various mutants for (**A**) D-ribose phosphorylation (2 mM) (**B**) D-arabinose phosphorylation (50 mM). Data represented in mean values and standard deviation (in three repeats).

Having selected substitutions feasible for investigation in vitro, we introduced these mutations in the gene encoding the wild-type *Eco*RK, created efficient producer strains and produced and purified the recombinant mutant enzymes (along with the wild type) to a purity of 85 ± 5% (Figure S1). We then performed the enzyme assay for the wild type and mutant *Eco*RK determining the ribose and arabinose phosphorylation activity through the detection of conversion of ATP to ADP via HPLC. For the natural substrate ribose, an enzyme assay was performed at an equimolar concentration (2 mM ribose, 2 mM ATP). Since the native *Eco*RK affinity towards arabinose is too low to perform enzyme assay at equimolar concentration, we used a 25-fold excess of arabinose (50 mM arabinose, 2 mM ATP). This concentration is close to the one we have used in our previous study, dealing with the preparative cascade synthesis of arabinosides using wild type enzymes [4]. The obtained results are presented in Figure 6.

The enzyme assay revealed that all of the investigated mutations decreased *Eco*RK activity towards the natural substrate ribose, with the double mutation demonstrating the most pronounced effect. This result confirms our hypothesis that Ala98, Ile100 and Ile110 residues form a hydrophobic interface that plays a role in ribose binding. Most importantly, the A98G substitution resulted in an increase in *Eco*RK activity towards arabinose by an order of magnitude. The double A98G/I100V mutation has also resulted in a significant positive effect, although the observed increase was less than that of the single A98G mutation. Meanwhile, I100V did not have any significant effect and I110V resulted in a fivefold decrease in enzyme activity. To conclude, we have determined that two *Eco*RK mutants display elevated activity towards D-arabinose, and therefore proceeded to the comparative study of enzyme kinetics.

We have determined the kinetic parameters for the enzymatic activity of the native enzyme, the single A98G and the double A98G/I100V mutants towards D-ribose, D-arabinose and ATP. The results are presented Table 1. Michaelis–Menten plots are presented in Figure S2.

Kinetic studies of the wild type enzyme have shown that arabinose has a two orders of magnitude lower k_cat_ and three orders of magnitude higher K_M_ compared to the natural substrate. Comparison of kinetic parameters illustrates that the A98G mutation caused opposite effects for *Eco*RK activity towards ribose and arabinose. In the case of ribose, this substitution resulted in a twofold increase in K_M_ and decrease in k_cat_, highlighting the importance of Ala98 for both natural substrate binding and catalysis. On the other hand, for our substrate of interest, D-arabinose, we observed a 15-fold increase in k_cat_ and 1.5-fold decrease in K_M_. The second I100V mutation has caused a slight decrease in k_cat_ and a twofold increase in K_M_ for arabinose phosphorylation compared to the A98G mutant. In terms of activity towards ATP, each of the two subsequent mutations caused a slight increase in K_M_.

To conclude, the single A98G mutant turned out to be the most active towards arabinose. With the achieved 15-fold increase in k_cat_ towards arabinose, our enzyme design project turned out to be fully successful. To illustrate the practical applicability of our results, we carried out a comparative study of the performance of the wild type *Eco*RK and A98G mutant in the cascade synthesis of arabinosides. For this purpose, we chose 2-chloroadenine arabinoside as the test subject, which is a compound closely related to the FDA-approved drugs cladribine (deoxyriboside) and clofarabine (2-deoxy-2-fluoroarabinoside) [24,25]. We have previously reported the cascade synthesis of 2-chloroadenine arabinoside with wild type *Eco*RK, phosphopentomutase and purine nucleoside phosphorylase from *E. coli* (*Eco*PPM and *Eco*PNP, respectively) [4]. We have performed the synthesis of 2-chloroadenine arabinoside using wild type and A98G mutant *Eco*RK in the same reaction conditions, although with a 10-fold reduction in the quantity of the mutant enzyme.

**Figure 7:**
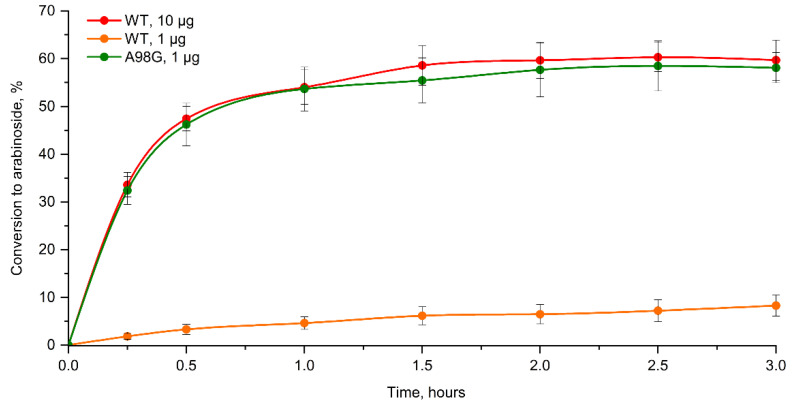
Accumulation of 2-chloroadenine arabinoside during cascade synthesis with the wild type *Eco*RK and the A98G mutant. The data are presented in mean values with standard deviation. Data represented in mean values and standard deviation (in three repeats).

As a result, the synthesis utilizing 1 μg of A98G mutant was as efficient as that with 10 μg of the wild type enzyme (Figure 7). In both cases, the conversion of 2-cloroadenine to the desired arabinoside product peaked at 60% after 1.5 h of incubation. Meanwhile, synthesis with only 1 μg of native *Eco*RK was highly inefficient: the conversion did not exceed 9% after 3 h. Therefore, we have proven that our engineered A98G mutant allows us to perform the cascade synthesis of arabinosides with an order of magnitude less enzyme quantity with no loss of synthesis efficiency.

## 3. Discussion

Our enzyme design project was dedicated to engineering a mutant form of *Eco*RK with elevated activity towards D-arabinose. Molecular dynamics simulations have allowed us to study the binding of arabinose by *Eco*RK in silico and hypothesize the reasons behind the low enzyme activity towards this unnatural substrate. Guided by our hypothesis, we have selected four mutations worthy of investigation in vitro: A98G, I100V, I110V and A98G/I100V. Among these mutations, A98G turned out to be the most efficient, resulting in an order of magnitude 15-fold increase in k_cat_ and 1.5-fold decrease in K_M_. Therefore, the target of our research was successfully achieved.

The order of magnitude increase in enzyme activity allows us to lower the amount of recombinant enzyme used in cascade synthesis, which is a major factor limiting the feasibility of the synthesis of arabinosides using wild type enzymes. We have also achieved a 1.5-fold decrease in K_M_, which can allow us to perform cascade synthesis using a lower concentration of arabinose. While the K_M_ value of arabinose phosphorylation by *Eco*RK A98G remains very high, the need for an excess of this substrate is not a critical issue, since D-arabinose is a commodity reagent.

We have illustrated the practical benefits of the A98G mutant through the cascade synthesis of 2-chloroadenine arabinoside as a test subject. Our previous research has shown that state-of-art wild type *Eco*RK requires large amounts of the recombinant enzyme for the efficient synthesis of arabinosides, making the feasibility of the cascade synthesis of these compounds highly questionable. However, the result of this research project, namely the A98G mutant, allows us to perform the cascade synthesis of 2-chloroadenine arabinoside with an order of magnitude less of the enzyme without a loss in substrate conversion. Therefore, with our engineered mutant, the narrow substrate specificity of ribokinase is no longer such a critical issue for the cascade synthesis of arabinosides. Our engineered *Eco*RK A98G mutant can be a basis for future research, dedicated to the development of efficient approaches of cascade synthesis of medically important cancer suppressing and antiviral nucleosides. The narrow carbohydrate substrate specificity of other enzymes of this cascade (namely PPM and PNP) remains a challenge that we are going to address in our future enzyme design projects.

Another aspect of our research that is worthy of discussion are the implications of our results for knowledge concerning the *Eco*RK catalytic mechanism, and the relationship between the enzymes structure and substrate specificity.

Both enzyme assay and kinetic studies of wild type and mutant *Eco*RK activity towards ribose confirm that the Ala98, Ile100 and Ile110 play an important role in the natural reaction. Molecular dynamics simulations have shown that atoms of these three residues are exposed to the non-polar 2′-hydrogen atoms of ribose (Figure 3 and Figure 4F). In context of this structural data, the negative effect of Ala→Gly and Ile→Val substitutions on *Eco*RK activity towards ribose can be explained by the disruption of an important hydrophobic interaction through a reduction in the sidechain size of Ala98, Ile100 and Ile110.

The study of arabinose binding in silico has revealed that this substrate assumes the  Twist23 conformation in the active site of *Eco*RK. The 2′-hydroxyl group of arabinose is exposed to the three mentioned lid-domain residues that form a hydrophobic interface. We have observed that throughout the MD simulation the β-carbon atom of Ala98 was the closest to Ara-2′OH. This fact can explain why A98G has caused an order of magnitude increase in k_cat_ towards arabinose, while I100V and I110V did not display any positive effect. The double A98G/I100V mutant had a slightly lower k_cat_ and a twofold higher K_M_ compared to the single A98G mutant, which further shows that the I100V had no positive effect on arabinose binding.

Since we have measured pentose phosphorylation through the detection of the conversion of ATP to ADP, we also had to take into account the possibility that the effects we have observed may also be caused by the influence of the investigated mutations on *Eco*RK activity towards ATP. Kinetic studies have shown that the A98G and A98G/I100V mutations have increased the K_M_ for ATP. Therefore, the observed positive effects of A98G and A98G/I100V mutations on this activity towards arabinose cannot be explained by a change in affinity of *Eco*RK towards the phosphate donor.

To conclude, we have successfully achieved all of the goals of our enzyme-design project. We have developed a mutant form of ribokinase (*Eco*RK A98G) with a 15-fold increase in k_cat_ and twofold decrease in K_M_ towards D-arabinose. We have shown that this mutant allows the cascade synthesis of arabinosides with an order of magnitude less enzyme with no loss in synthesis efficiency. Our mutational studies provide insight into the roles of three hydrophobic residues of the lid domain (Ala98, Ile100 and Ile110) in the substrate specificity of *Eco*RK, and, in particular, explain one of the reasons behind the enzyme’s low affinity towards arabinose.

## 4. Materials and Methods

### 4.1. Computational Approaches

The analysis, visualization and editing of molecular models were performed in PyMOL 2.0 (Schrödinger, LLC, New York, NY, USA). Simulations of molecular dynamics were carried out using the Gromacs 2021 package modified with PLUMED v2.8.0 plugin [26,27]. The parameters of the molecular models were described using Amber ff99SB-ILDN forcefield [28]. Ligand parameters were obtained through the Antechamber package (AmberTools2022) using the bcc charge calculation method [29].

The structure of the complex between *Eco*RK and its natural substrate ribose was fetched from the Protein Data Bank (PDB 1RKD). The inversion of the chirality of the C2′ atom of ribose in this structure via molecular editing was performed to obtain the model of the enzyme in a complex with arabinose (initial approximation). Both of the protein-ligand complexes were placed into the centers of rhombic dodecahedral periodic cells (1000 nm^3^ in volume, so that any protein atom was at least 1 nm away from the cell edge), filled with tip3p water-molecule models. Systems were neutralized with KCl ions to a concentration of 150 mM.

The obtained systems were subjected to energy minimization via the steepest descent method until no atom was subject to a force larger than 1000 kJ/(M∙nm^−2^). The next step was a 0.5-ns long NVT equilibration for 333,15 K using a modified Berendsen (V-rescale) thermostat, followed by 0.5-ns long NPT equilibration for 1 bar using the Parrinello–Rahman barostat [30,31].

Productive molecular dynamics simulations were carried out in an isothermal-isobaric ensemble with the same thermostat and barostat as used during equilibration. Integration with a time step of 2 fs was carried out using the Leap-Frog algorithm [32]. The non-covalent interactions were only taken into account for atoms within 1.4 nm of each other. We used the smooth particle mesh Ewald (PME) summation method with cubic interpolation and grid spacing in Fourier to account for the long-range electrostatic interactions [33]. The bonds were constrained using the LINCS algorithm [34].

We performed productive MD simulations, resulting in two 400-ns long trajectories. These trajectories were modified via the gmx trjconv command with the –pbc flag so that no molecule was split due to periodic boundary conditions. RMSD, RMSF and gyration radius were calculated using the gmx rms, gmx rmsf and gmx gyrate commands, respectively. Distance between atoms and sugar pseudorotation coordinates were calculated via the PLUMED plugin.

### 4.2. Genetic Engineering and Producer Strain Cultivation

We have used the plasmid vector from our previous research (Chuvikovsky et al., 2006), containing the rbsK gene, encoding ribokinase from *E. coli* (Uniprot P0A9J6), [15]. Mutations in the rbsK gene were performed using the Phusion Site-Directed Mutagenesis Kit (Thermo Fisher Scientific, Waltham, MA, USA). The obtained expression vectors (along with the one containing the native gene) were transformed into competent *E. coli* ER2566 (New England Biolabs, Ipswich, MA, USA) strain cells.

The resulting producer strains were cultivated in the Luria–Bertani medium (per 1 L: 10 g. tryptone, 5 g. yeast extract 10 g. NaCl) with 100 µg/mL ampicillin. Cell culture was cultivated until reaching A595 = 0.8, followed by supplementation with 0.4 mM IPTG (isopropyl β-D-1-thiogalactopyranoside). After induction by IPTG, the cells were cultivated for 4 h at 37 °C, resulting in recombinant enzyme in soluble form.

### 4.3. Purification of EcoRK

The cells were resuspended in 0.1 M Tris-HCl pH 8, 1 mM PMSF (phenylmethylsulfonyl fluoride) buffer (1:10 *w*/*v*) and homogenized to ultrasonic disintegration, followed by centrifugation. Cell supernatant was diluted with three volumes of distilled water and applied onto the XK16/10 (GE Healthcare, Chicago, IL, USA) column, packed with 5 mL Q Sepharose High Performance sorbent, pre equilibrated with 25 mM Tris–HCl and 5 mM EDTA buffer. After washing the column, the recombinant enzyme was eluted with a linear 0–0.5 M NaCl gradient.

The fractions containing the desired recombinant protein were pooled, concentrated to 10 ± 2 mg/mL on the YM-30 membrane (Millipore, Burlington, MA, USA) and applied onto the Superdex 200 column 16 × 70 mm (Amersham Bioscience, Amersham, UK), pre-equilibrated with the final buffer: 50 mM Tris-HCl pH8, 50 mM KCl. 5 mM MgCl_2_, 5% glycerol, 0.04% NaN_3_. The eluted purified protein was used for further experiments. We used the Bradford method to determine protein concentration and polyacrylamide gel electrophoresis under denaturating conditions for the determination of protein purity [35,36].

### 4.4. Enzyme Assay and Determination of Kinetic Parameters

The 100 μL reaction mix for the enzyme assay included: 2 mM D-ribose or 50 mM D-arabinose, 2 mM ATP, 50 mM Tris-HCl pH8, 50 mM KCl, 5 mM MgCl_2_, 1 mM KH_2_PO_4_. The samples were pre-heated, followed by the addition of the investigated enzyme in a quantity of 1.5 ×·10^−2^ μg for ribose and 1 μg for arabinose. Samples supplemented with the studied enzyme were incubated for four minutes at 37 °C, after which an enzyme assay was performed via analytical HPLC (in three repeats).

The conversion of the substrate into the product was observed through the detection of the ATP and ADP via HPLC. Analytical HPLC was performed on the Waters system (pump 1525, detector 2489, Breeze Software, Milford, NH, USA) with the following method: Supelcosil LC-18-T, 5 µm, 150 × 4.6 mm column, isocratic elution with 100 mM KH_2_PO_4_ pH6, flow rate 0.4 mL/min, detection at 254 nm. One unit of *Eco*RK activity was defined as 1 μmol of pentose substrate converted into product per minute per milligram of recombinant protein.

The reaction mix (100 μL) for the determination of the kinetic parameters of ribose phosphorylation included: 0.06–6 mM D-ribose, 2 mM ATP, 50 mM Tris-HCl pH8, 50 mM KCl, 5 mM MgCl_2_, 1 mM KH_2_PO_4_ and 1.5 × 10^−2^ μg of the investigated enzyme. The reaction mix (100 μL) for arabinose included: 50–1600 mM D-arabinose, 2 mM ATP, 50 mM Tris-HCl pH8, 50 mM KCl, 5 mM MgCl_2_, 1 mM KH_2_PO_4_ and 1 μg of wild type *Eco*RK or 0.15 μg of the mutant enzyme. The reaction mix (100 μL) for the determination of kinetic parameters with ATP as the variable substrate included: 0.1–5 mM ATP, 2 mM D-ribose, 50 mM Tris-HCl pH8, 50 mM KCl, 5 mM MgCl_2_, 1 mM KH_2_PO_4_ and 1.5 × 10^–2^ μg of the investigated enzyme. Enzyme assay for the varying substrate concentrations were performed as described above. We determined the kinetic parameters through nonlinear regression analysis using the SciDAVis v2.3.0 program. Catalytic constants were calculated for one enzyme subunit (32.3 kDa according to amino acid sequence). The enzyme kinetics model was fitted to the Michaelis–Menten equation.

### 4.5. Cascade Synthesis of 2-Chloroadenine Arabinoside

The reaction mix (500 μL) for the cascade synthesis of 2-chloroadenine arabinoside included: 60 mM D-arabinose, 0.34 mM 2-chloroadenine; 2.5 mM ATP; 50 mM Tris-HCl pH8, 2.5 mM MnCl_2_, 50 mM KCl, 2 mM KH_2_PO_4_, ribokinase from *E. coli* (1 or 10 µg of the wild type enzyme, or 1 µg of the A98G mutant), 200 µg of phosphopentomutase from *E. coli* and 40 µg of purine nucleoside phosphorylase from *E. coli*. Recombinant phosphopentomutase from *E. coli* and purine nucleoside phosphorylase from *E. coli* were obtained in sufficient quantities according to the methods provided in their respective publications [10,37].

The reaction mixes with the wild type and A98G mutant *Eco*RK were incubated at 50 °C for 3 h. The conversion of 2-chloroadenine into 2-chloroadenine arabinoside was detected through analytical HPLC on the Waters system (Waters 1525, Waters 2489, Breeze) in three repeats with the following method: column YMC Triart-C18, 50 × 3.0 mm, 3 µm, eluent 0.1% aqueous trifluoroacetic acid and 7% acetonitrile, detection at 254 nm, flow rate 0.4 mL/min.

## Figures and Tables

**Table 1:** Kinetic parameters for the enzymatic activity of wild type EcoRK, A98G and A98G/I100V mutants towards D-ribose, D-arabinose and ATP (determination of activity was performed in three repeats).

	V_max_, μmol/min·mg	k_cat_, s^−1^	K_M_, mM
D-ribose:			
Wild type	340 ± 50	180 ± 30	0.15 ± 0.02
A98G	180 ± 20	97 ± 11	0.28 ± 0.03
A98G/I100V	130 ± 30	70 ± 16	0.24 ± 0.05
D-arabinose:			
Wild type	3.6 ± 0.5	1.9 ± 0.3	470 ± 60
A98G	54 ± 5	29 ± 3	310 ± 30
A98G/I100V	33 ± 5	17 ± 3	730 ± 120
ATP:			
Wild type	350 ± 40	185 ± 21	0.43 ± 0.05
A98G	160 ± 10	85 ± 5	0.58 ± 0.04
A98G/I100V	120 ± 10	63 ± 5	0.75 ± 0.09

## Data Availability

The data presented in this study are available on request from the corresponding author.

## Supplementary Materials

The following supporting information can be downloaded at: https://www.mdpi.com/article/10.3390/ijms232012540/s1.
