# Supplementary material for: Rational Mutagenesis in the Lid Domain of Ribokinase from E. coli Results in an Order of Magnitude Increase in Activity towards D-arabinose
Source: Int J Mol Sci. 2022 Oct 19;23(20):12540. doi: 10.3390/ijms232012540 (PMC9604405; doi:10.3390/ijms232012540)
Supplement: Supplementary file 1 [file ijms-23-12540-s001.zip › ijms-1931923-supplementary.pdf]

Supplementary material

Rational Mutagenesis in the Lid Domain of Ribokinase from E. coli Results in an Order of Magnitude Increase in Activity towards D-arabinose

Evgeniy A. Zayats*, Ilya V. Fateev, Maria A. Kostromina, Yulia A. Abramchik, Dmitry D. Lykoshin, Daria O. Yurovskaya, Vladimir I. Timofeev, Maria Ya. Berzina, Barbara Z. Eletskaya, Irina D. Konstantinova* and Roman S. Esipov

Shemyakin and Ovchinnikov Institute of Bioorganic Chemistry, Russian Academy of Sciences, Miklukho-Maklaya St. 16/10, 117997, Moscow, Russia

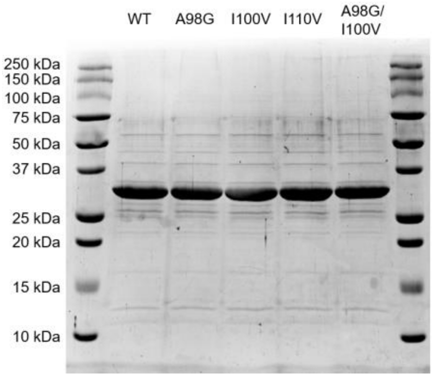

Figure S1. SDS PAGE analysis of the wild type *Eco*RK and the four investigated mutant forms (5 µg of protein per lane). Marker - Precision Plus Protein All Blue Standard, Biorad.

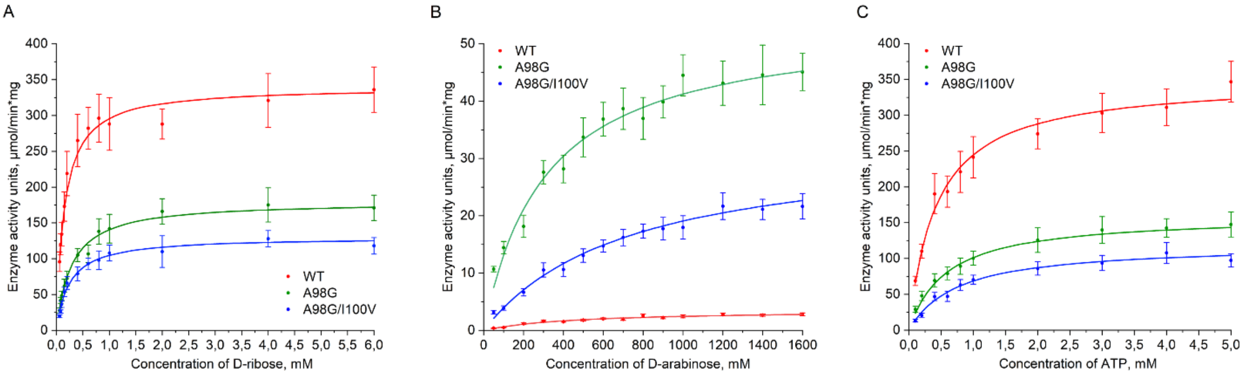

Figure S2. Michaelis-Menten plots for the enzymatic activity of wild type *Eco*RK, A98G and A98G/I100V mutants towards D-ribose (A), D-arabinose (B) and ATP (C). Data represented in mean values and standard deviation (three repeats).
